# A meta-synthesis of qualitative studies on cardiovascular disease patients’ experiences using digital health tools

**DOI:** 10.3389/fpubh.2025.1709562

**Published:** 2025-11-10

**Authors:** Yuxuan Shao, Xinyi Hou, Qin Peng, Mengdie Hu, Chaofeng Li

**Affiliations:** 1 School of Nursing, Nanjing University of Chinese Medicine, Nanjing, China; 2 Department of Cardiology, The Second Hospital of Nanjing, Affiliated to Nanjing University of Chinese Medicine, Nanjing, China

**Keywords:** cardiovascular diseases, telehealth, self-management, qualitative study, meta-synthesis, evidence-based nursing science

## Abstract

**Objectives:**

This review aims to provide deeper insights into the patient experience by synthesizing qualitative findings from studies on DHTs used in CVD care. Specifically, it seeks to answer the question: What are the key barriers, facilitators, and trends in the use of digital interventions for CVD rehabilitation and self-management? Unlike traditional quantitative reviews, this study focuses on understanding the human factors, personal narratives, and contextual influences that shape the uptake and impact of DHTs in this area.

**Methods:**

A qualitative meta-synthesis was conducted on articles from the following major electronic databases: PubMed, Cochrane Library, CINAHL, Web of Science, Embase, PsycINFO, and Chinese databases, including Chinese National Knowledge Infrastructure (CNKI), Wanfang Database (CECDB), VIP Database, and China Biomedical Database (CBM). Studies published between 2020 and 2025 were included, and a systematic search was conducted using predefined keywords. Papers were selected based on predefined inclusion and exclusion criteria. The synthesis employed the aggregative integration approach from the meta-synthesis method proposed by the Joanna Briggs Institute (JBI) Evidence-based Healthcare Center in Australia. The extracted qualitative data were compared and analyzed in-depth.

**Results:**

A total of 21 studies were included; 92 findings were extracted, organized into 13 categories, and consolidated into five synthesized findings: positive effects of DHTs; barriers to adoption and sustained use of DHTs; optimization needs and design priorities for DHTs; and culture- and disease-specific needs.

**Conclusion:**

Cardiovascular DHTs should be grounded in patients’ individualized needs and operationalized through age-friendly technological innovations, integration of professional support, and culturally sensitive design. Leveraging multiple pathways and modalities to strengthen the family–hospital–community support system will improve the acceptability of DHTs among people with cardiovascular disease. What this review uniquely adds is the patient-reported affective/relational value (e.g., feeling “continuously accompanied,” increased reassurance and trust through human-in-the-loop supervision) and the contextual value of digital health (age-friendly, culturally and disease-specific tailoring integrated with family/clinician support).

## 1 Introduction

Cardiovascular diseases (CVD) remain the leading cause of death and disability worldwide, and the global burden continues to grow. According to the World Heart Federation’s World Heart Report 2023 (1), CVD caused 20.5 million deaths in 2021—approximately one-third of all global deaths—with 80% occurring in low- and middle-income countries (LMICs). If current trends persist, CVD deaths could rise to 35.6 million by 2050 (2). In China, CVD likewise ranks first among causes of death in both urban and rural populations. The China Cardiovascular Health and Disease Report 2023 (3, 4) estimates that approximately 330 million people are living with CVD—nearly one quarter of adults. In 2021, CVD accounted for more than 40% of all deaths, with higher prevalence in Northeast and North China than in South China (5), underscoring the severity of the burden and regional inequities in prevention and control.

Against this backdrop, digital health tools (DHTs) are a pivotal pathway for optimizing patient management and reducing inequities in resource distribution and access to care. Representative tools—telerehabilitation, mobile health (mHealth) applications, and wearable devices—offer several advantages: continuous remote monitoring, enhanced self-management, strengthened clinician–patient collaboration, and potential gains in cost and efficiency. They show particular promise for improving access and continuity of care in primary care and resource-limited settings (6). Growing evidence (7–9) shows that DHTs improve process indicators such as medication adherence, self-monitoring frequency, and appointment attendance in secondary prevention. These tools have also been shown to improve clinical outcomes like blood pressure control and lipid management. For example, mHealth strategies using interactive voice response (IVR) and text messaging strengthen risk-factor management, facilitate blood-pressure and lipid control, and reduce heart-failure readmissions (10, 11). Wearable devices (e.g., smartwatches, ECG patches) support continuous rhythm surveillance and report positive predictive values of 84–98% for detecting atrial fibrillation, enabling earlier detection and intervention (12). Relevant qualitative study (13) further indicate that patients’ experiences include a sense of being “continuously accompanied” and emotionally supported, as well as identity affirmation, relational trust, informational transparency, perceived privacy, and burden. However, while several studies have explored the impact of DHTs, there is a lack of cross-study synthesis that clarifies how patients experience these tools, why they adopt or resist them, and under what conditions they are effective. Existing qualitative syntheses (14) have examined barriers and facilitators in cardiac rehabilitation, but there is still a gap in understanding the holistic experience of cardiovascular patients with DHTs, particularly in varied settings and across diverse patient populations.

Moreover, individuals with cardiovascular disease are typically older, have multiple comorbidities, and vary widely in functional status (15). These features heighten usability requirements, increase perceived burden, and demand higher levels of health literacy and digital health literacy (16). Differences across the digital health ecosystem—platforms (apps, mini-programs, wearables, telehealth portals), use contexts (post-discharge rehabilitation, community follow-up, home self-management), and health-system levels (primary care, specialty care, disease-specific programs)—produce substantial heterogeneity in patient experience and intervention effects (17, 18). This meta-synthesis aims to fill this gap by providing an integrative and comprehensive understanding of the barriers, facilitators, and optimization needs in DHTs for CVD patients. Unlike prior reviews that primarily emphasized technical efficacy, our meta-synthesis foregrounds patient-reported affective/relational value and contextualized design needs, showing how professional support and culture−/disease-specific tailoring convert data into safety, trust, and sustained action.

## 2 Methods

### 2.1 Inclusion and exclusion criteria

#### 2.1.1 Study design (S)

The qualitative research studies from which qualitative data could be extracted, the primary qualitative research studies were included but were not limited to methodologies, such as phenomenology, grounded theory, action research, ethnography, and feminist research.

#### 2.1.2 Participant (P)

Patients with CVD as defined by the World Health Organization (WHO).

#### 2.1.3 Interest of phenomena (I)

Patients’ experiences with DHTs (e.g., mobile health applications, remote monitoring, and wearables), encompassing acceptance, barriers, and facilitators.

#### 2.1.4 Context (Co)

We will exclude studies without accessible full text, duplicate publications, articles not published in Chinese or English, and mixed-methods studies in which the qualitative component cannot be extracted for synthesis.

### 2.2 Design

This study adopts an exploratory qualitative meta-synthesis design to identify, appraise, and synthesize primary qualitative evidence on CVD patients’ experiences of using DHTs (e.g., mHealth apps, telerehabilitation platforms, remote monitoring, and wearables). This review aims to generate higher-order, transferable insights to inform the patient-centered redesign and implementation of DHTs in CVD care. Reporting will follow PRISMA guidance for qualitative evidence syntheses. A meta-synthesis approach was used to combine and present the qualitative findings (19). Inspired by Sandelowski et al. (20), meta-synthesis of qualitative research is based on the premise of understanding its philosophical thoughts and methodology, repeatedly reading the included literature and extracting the themes and hidden meanings so as to conduct inductive analysis, form new categories, and finally integrate new results. By synthesizing new results, a more profound and substantial explanation can be given to specific phenomena, creating new perspectives and so-called “third-level” findings, providing a more influential and persuasive final conclusion. Relevant articles were searched, and data were extracted and critically evaluated using a thematic synthesis based on the three steps outlined by Thomas and Harden (21), i.e., text coding line by line, developing descriptive themes, and generating analytical themes. This study met the requirements of the Helsinki Declaration.

### 2.3 Search methods

To ensure adequate performance in searches (i.e., recall, precision, and number needed to read), we selected a combination of 11 databases for the literature search of systematic reviews, including both Chinese and English databases that are widely used in the field of health care. Qualitative studies published in PubMed, Cochrane Library, CINAHL, Web of Science, Embase, CINAHL, PsycINFO and Chinese databases, including Chinese National Knowledge Infrastructure (CNKI), Wanfang Database (CECDB), VIP Database, and China Biomedical Database (CBM) from January 2020 to June 2025 were searched by two researchers (YS and XH) in July 2025. The search terms were developed, and subject headings were used where possible and adjusted for different databases. Four groups of keywords or MeSH terms were included and combined using Boolean operators: (1) heart*, heart disease*, heart disorder*, cardiac*, cardiovascular, coronary heart disease*, myocardial infarction, acute coronary syndrome, angina, stable, heart failure, arrhythmias, cardiac, cardiac insufficiency, valvular heart disease*, aortic dissection, congenital heart disease*, hypertension; (2) Telemedicine, Mobile Applications, Wearable Electronic Devices, Digital Health, mHealth, eHealth, health app*, smartwatch, remote monitor*; (3) Patient Acceptance of Health Care, “Attitude to Health OR Health Knowledge, Attitudes, Practice,” acceptab*, barrier*, facilitator*, adherence, user experience, perception*; (4) Qualitative Research, Focus Groups, Interviews as Topic, Narratives, Ground Theory, phenomenolog*, ethnograph*, thematic analysis, semi-structured interview*, lived experience. To determine the eligibility of the potentially relevant studies, all titles and abstracts were reviewed by researchers. Box 1 showed the search strategy with PubMed as an example.

**BOX 1** Search strategy in PubMed.

#1 (("Myocardial Infarction"[Mesh]) OR "Acute Coronary Syndrome"[Mesh] OR "Angina, Stable"[Mesh] OR "Heart Failure"[Mesh] OR "Arrhythmias, Cardiac"[Mesh] OR "Aortic Dissection"[Mesh] OR (heart*[Title/Abstract] OR heart disease*[Title/Abstract] OR heart disorder*[Title/Abstract] OR cardiac*[Title/Abstract] OR cardiovascular[Title/Abstract] OR coronary heart disease*[Title/Abstract] OR cardiac insufficiency[Title/Abstract] OR valvular heart disease*[Title/Abstract] OR congenital heart disease*[Title/Abstract] OR hypertension[Title/Abstract]))

#2 ((Telemedicine[MeSH Terms] OR Mobile Applications[MeSH Terms] OR Wearable Electronic Devices[MeSH Terms] OR Digital Health[Title/Abstract] OR mHealth[Title/Abstract] OR eHealth[Title/Abstract] OR health app*[Title/Abstract] OR smartwatch[Title/Abstract] OR remote monitor*[Title/Abstract]))

#3 (("Patient Acceptance of Health Care"[Mesh] OR "Attitude to Health"[Mesh] OR "Health Knowledge, Attitudes, Practice"[Mesh] OR acceptab*[Title/Abstract] OR barrier*[Title/Abstract] OR facilitator*[Title/Abstract] OR adherence[Title/Abstract] OR user experience[Title/Abstract] OR perception*[Title/Abstract]))

#4 (("Qualitative Research"[Mesh] OR "Focus Groups"[Mesh] OR "Interviews as Topic"[Mesh] OR "Narration"[Mesh] OR Ground Theory[Title/Abstract] OR phenomenolog*[Title/Abstract] OR ethnograph*[Title/Abstract] OR thematic analysis[Title/Abstract] OR semi-structured interview*[Title/Abstract] OR lived experience*[Title/Abstract]))

#5 #1 AND #2 AND #3 AND #4

Filters: 2020–2025

Search results:165

### 2.4 Search outcomes

**Figure 1:**
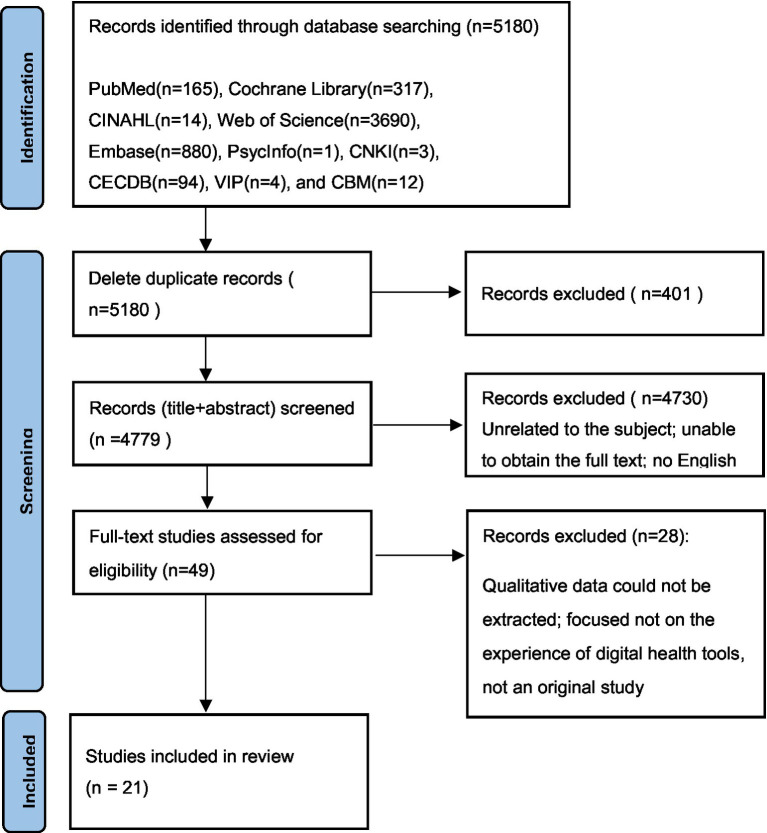
PRISMA flowchart.

Two researchers independently screened and extracted the literature according to the inclusion and exclusion criteria. An initial search using the above strategy yielded a total of 5,180 articles. First, the titles and abstracts of the articles were read to exclude those unrelated to the subject, were repetitive, and full text could not be obtained. Subsequently, 5,131 articles were excluded. After reading the full texts, 28 articles were excluded, and finally, 21 articles were identified as relevant. Also, no articles were traced from references. This search process is illustrated in Figure 1.

### 2.5 Quality appraisal

Two researchers independently assessed the methodological quality of the 21 included studies. Initially, the authors worked independently using the Joanna Briggs Critical Assessment Tool for Methodological Quality Assessment (22). This evaluation tool is widely applicable in the appraisal of qualitative research. The quality appraisal of research mainly focuses on the internal authenticity of the research, that is, the degree to which the research results are close to the true value, in order to determine whether there is bias. The evaluation tool consists of 10 questions designed to quickly and efficiently evaluate the studies with a simple yes, no, or unclear to each question. Each criterion was allocated a score (Yes = 2, No = 0, Unclear = l), giving a total score of 20 for each study. These scores were then converted to a percentage. Subsequently, the results were discussed to reach a consensus, studies with a score of more than 70% were considered acceptable after quality appraisal, otherwise they were excluded.

### 2.6 Data extraction

A comprehensive study was conducted to characterize the quality of the content and assess the methodological development in the collected studies (23). The extracted data included the author, the year of publication, country or region, research method, research subjects, interesting phenomena, and main research results. The results were cross-reviewed by two investigators, and any disagreement was resolved by discussion with a third investigator.

### 2.7 Data analysis and synthesis

We used meta-aggregation to synthesize the findings of the qualitative studies (24). This method of systematic review involves categorizing and re-categorizing the synthesized findings of two or more studies. First, each selected article was read several times to increase the understanding of research objectives, methods, and conclusions. Next, the results of each study are extracted, along with the research results showing that the results of data and text. The consistency between the research results and supporting data was assessed by two researchers independently, each finding provided its own level of credibility (23). For qualitative data, there are three levels of credibility: (1) Unequivocal (U): relates to evidence beyond a reasonable doubt, which may include findings that are matter of fact, directly reported/observed and not open to challenge; (2) Credible (C): those that are, albeit interpretations, plausible in light of data and the theoretical framework, They can be logically inferred from the data; (3) Not Supported (NS): when 1 nor 2 apply and when most notable findings are not supported by the data. Results were then encoded according to their meaning and content. Researchers looked for similarities and differences between the findings and the textual data, and the meanings of the original data set were classified. For each theme, when needed, sub-themes were also developed following the same process. Finally, these categories were repeatedly assessed to identify the similarities and obtain synthesized results.

### 2.8 Ethical considerations

This meta-analysis was carried out in compliance with the Preferred Reporting Items for Systematic reviews and Meta-Analyses (PRISMA) guidelines and referred only to published data. Therefore, no ethical approval was required. However, ethical approval in the included empirical qualitative studies were assessed.

## 3 Results

**Figure 2:**
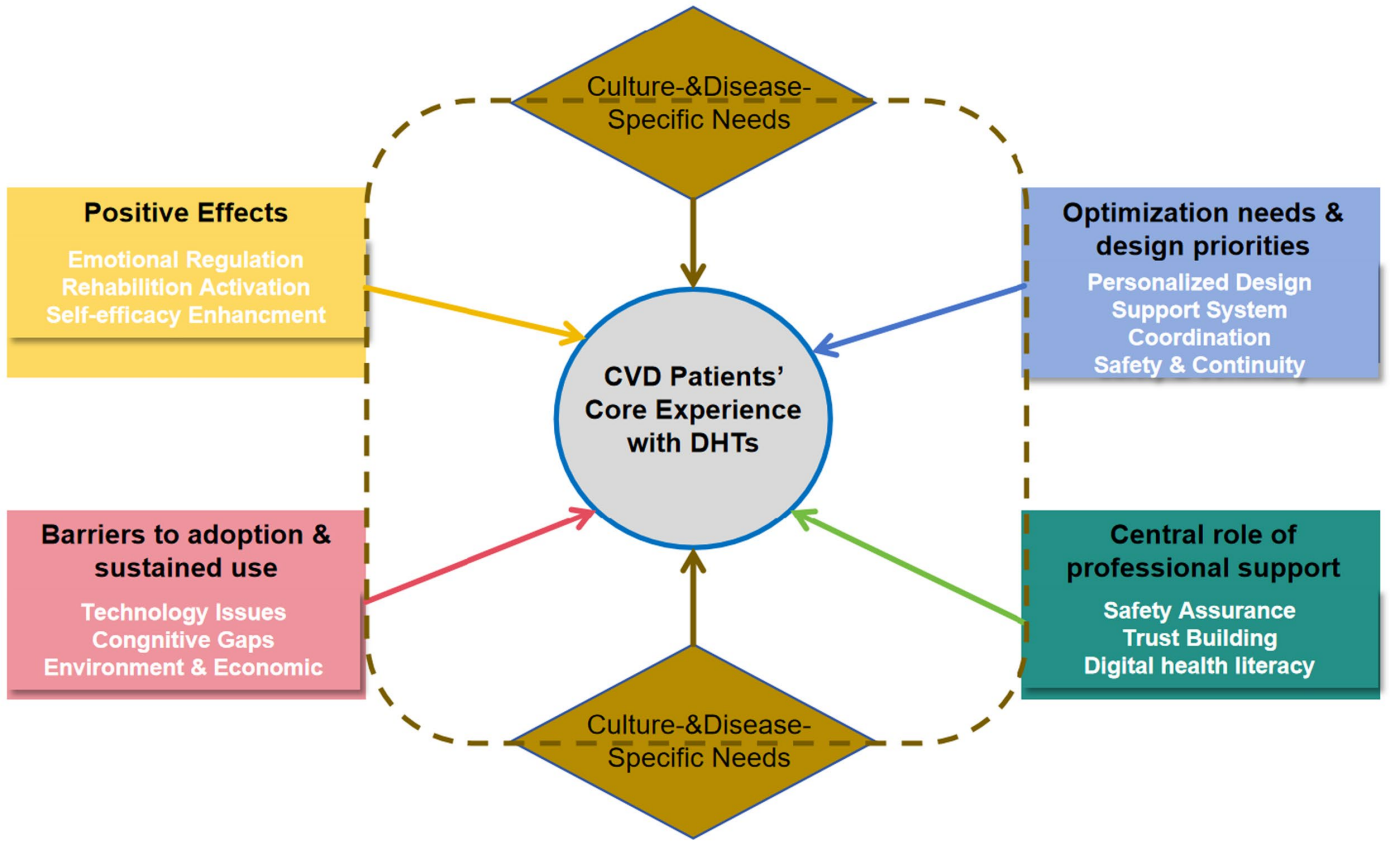
Conceptual model.

The 21 studies (25–45) were conducted in the following countries: China (*n* = 4), USA (*n* = 4), Italy (*n* = 1), Sweden (*n* = 2), Ireland (*n* = 1), Canada (*n* = 1), Australia (*n* = 2), Singapore (*n* = 1), Korea (*n* = 2), Chinese Hong Kong (*n* = 1), Chinese Macao (*n* = 1) and Germany (*n* = 1). These studies involved 442 CVD patients. Research methods included: phenomenological approaches (*n* = 12), descriptive qualitative analyses (*n* = 8) and grounded theory (*n* = 1). All studies were published after 2020 and were original articles. The results of the literature quality appraisal were shown in Table 1, the extraction results were presented in Table 2. The PRISMA was used as a basis for the results of syntheses. Five major themes emerged from the selected studies, reflecting the real experience of CVD Patients’ experiences of using DHTs. These themes were: Positive effects of DHTs, Barriers to adoption and sustained use of DHTs, Optimization needs and design priorities for DHTs, Central role of professional support in DHTs implementation, Culture- and disease-specific needs. The themes were divided into several sub-themes of meaningful units, as demonstrated in Table 3. The dynamic relationships between these five synthesized findings are illustrated in a conceptual model (Figure 2), which positions patient-centered DHI implementation as the ultimate goal influenced by the other four themes.

**Table 1:** Methodological quality appraisal of included qualitative studies using the JBI checklist.

Reference*	Q1**	2	3	4	5	6	7	8	9	10	Result
Lan Chunhan et al. (41)	Y	Y	Y	Y	Y	N	N	Y	Y	Y	18/20 (90%)
Liu Shenxinyu et al. (31)	Y	Y	Y	Y	Y	N	Y	Y	Y	Y	19/20 (95%)
Tang Cong et al. (39)	U	Y	Y	Y	Y	N	Y	Y	Y	Y	18/20 (90%)
Micheluzzi et al. (26)	U	Y	Y	Y	Y	N	Y	Y	Y	Y	18/20 (90%)
Nilsson et al. (36)	Y	Y	Y	Y	Y	Y	Y	Y	Y	Y	20/20 (100%)
O’Shea et al. (44)	Y	Y	Y	Y	Y	N	N	Y	Y	Y	18/20 (90%)
Sivakumar et al. (35)	Y	Y	Y	Y	Y	N	Y	Y	Y	Y	19/20 (95%)
Su et al. (37)	Y	Y	Y	Y	Y	N	Y	Y	Y	Y	19/20 (95%)
Trivedi et al. (29)	U	Y	Y	Y	Y	N	N	Y	Y	Y	17/20 (85%)
Yang et al. (28)	U	Y	Y	Y	Y	N	Y	Y	Y	Y	18/20 (90%)
Cher et al. (42)	U	Y	Y	Y	Y	N	U	Y	Y	Y	17/20 (85%)
Son et al. (43)	U	Y	Y	Y	Y	N	U	Y	Y	Y	17/20 (85%)
Blomqvist et al. (25)	Y	Y	Y	Y	Y	N	U	Y	Y	Y	18/20 (90%)
Choo et al. (27)	Y	Y	Y	Y	Y	N	U	Y	Y	Y	18/20 (90%)
Chu et al. (32)	Y	Y	Y	Y	Y	N	U	Y	Y	Y	18/20 (90%)
Foreman et al. (45)	Y	Y	Y	Y	Y	N	U	Y	Y	Y	18/20 (90%)
Islam et al. (30)	U	Y	Y	Y	Y	N	U	Y	Y	Y	17/20 (85%)
Lao et al. (38)	Y	Y	Y	Y	Y	N	N	Y	Y	Y	18/20 (90%)
Madujibeya et al. (34)	U	Y	Y	Y	Y	N	Y	Y	Y	Y	18/20 (90%)
Neumann et al. (33)	Y	Y	Y	Y	Y	Y	N	Y	Y	Y	19/20 (95%)
Schmaderer et al. (40)	Y	Y	Y	Y	Y	N	Y	Y	Y	Y	19/20 (95%)

*Critical appraisal (*n* = 10) of (Y = yes; N = no; U = unclear; NA = not applicable). **Question = Q.

**Table 2:** Characteristics of included studies (*n* = 21): country/region, method, participants, aims, and key findings.

References	Country	Research method	Participant	Aim	Results
Lan Chunhan et al. (41)	China	Qualitative descriptive study; semi-structured interviews	11 heart-failure patients who discontinued a mobile medical app mid-course	To explore real-world experiences and reasons for discontinuation among patients with low willingness to use an HF mHealth app	Five themes: (i) sociodemographic characteristics influence willingness to use; (ii) personal innovativeness influences willingness; (iii) low perceived ease of use reduces willingness; (iv) limited perceived usefulness reduces willingness; (v) negative psychological experiences reduce willingness
Liu Shenxinyu et al. (31)	China	Qualitative descriptive study; semi-structured interviews	17 hospitalized HF patients	To examine willingness and needs regarding a cardiac-rehab app and analyzed potential influencing factors	Four themes: (i) divergent patient attitudes; (ii) shifting needs for health information; (iii) in-app attributes and feature configuration; (iv) influences from the broader information environment
Tang Cong et al. (39)	China	Phenomenological approach; semi-structured interviews	13 patients with hypertension	To explore users’ experience with a mobile medical platform	Five themes: (i) changes in disease management and health behaviors; (ii) varied perceptions of sustained use; (iii) identification and endorsement; (iv) difficulties and concerns; (v) suggestions for improvement
Micheluzzi et al. (26)	Italy	Phenomenological approach; semi-structured interviews	22 home-dwelling HF patients	Experiences with immersive virtual reality during cardiac rehabilitation	Four themes: (i) cognitive and emotional benefits of iVR; (ii) impact on rehabilitation; (iii) customizable interventions; (iv) negative experiences with iVR
Nilsson et al. (36)	Sweden	Phenomenological approach; semi-structured interviews	15 post-MI cardiac-rehab patients	Perceptions of exercise-based cardiac telerehabilitation	Four themes: (i) crucial role of physiotherapists with specialist expertise; (ii) importance of prerequisites for willingness to participate; (iii) making exercise accessible and adjustable; (iv) motivating future exercise
O’Shea et al. (44)	Ireland	Phenomenological approach; semi-structured interviews	44 patients with CVD	Views and experiences of an eHealth cardiac-rehabilitation intervention (PATHway)	Five themes: (i) feedback on PATHway components; (ii) motivation; (iii) barriers to use; (iv) facilitators; (v) post-program reflections
Sivakumar et al. (35)	Canada	Qualitative descriptive study; focus groups	19 HF patients	Needs, motivations, and challenges in using mobile apps to support HF self-management	Four themes: (i) factors influencing technology use; (ii) support via information access and self-monitoring; (iii) fostering connection and communication; (iv) preferences for app features
Su et al. (37)	Chinese Hong Kong	Qualitative descriptive study; semi-structured interviews	20 patients with CHD	Experiences of a nurse-led eHealth cardiac-rehabilitation (NeCR) program	Five themes: (i) NeCR promotes behavior change; (ii) alleviates post-CHD emotional distress; (iii) shapes cognitive determinants; (iv) provides social support; (v) challenges encountered by patients
Trivedi et al. (29)	Australia	Phenomenological approach; semi-structured interviews	30 patients with atrial fibrillation	To explore perceptions of a voice-based conversational-AI support program	Four themes: (i) interactions with a voice-based conversational-AI program; (ii) engagement shaped by personalization, delivery mode, and frequency; (iii) improved AF care and access to information; (iv) empowerment for better AF self-management
Yang et al. (28)	China	Qualitative descriptive study; semi-structured interviews	20 post-MI patients	To explore experiences and perceptions of an AI large-language-model tool with personal health-record functionality (iflyhealth)	Four themes: (i) user engagement and experience; (ii) perceived benefits; (iii) challenges and barriers to use; (iv) reasons for refusal or discontinuation
Cher et al. (42)	Singapore	Phenomenological approach; semi-structured interviews	11 patients with AF and 12 healthcare providers	To explore attitudes toward using electronic tools for AF self-management and the factors shaping those attitudes	Eight themes: (i) current self-care strategies; (ii) patient characteristics and attitudes toward self-care; (iii) family and social support; (iv) support from hospitals; (v) perceived usefulness; (vi) technical preferences for e-tools; (vii) attitudes toward using e-tools; (viii) re-defining the role of non-electronic tools
Son et al. (43)	Korea	Phenomenological approach; semi-structured interviews	20 patients with chronic HF	To explore needs and views regarding using mHealth technologies at home	Four themes: (i) need for reliable and personalized health information; (ii) valuable features of mobile apps; (iii) barriers to adopting mHealth services; (iv) anticipated benefits of using mHealth technologies
Blomqvist et al. (25)	Sweden	Phenomenological approach; semi-structured interviews	10 patients with chronic HF	To describe experiences using an mHealth tool designed to support physical activity	Two themes: (i) cultivating awareness toward engaging in physical activity; (ii) motivation derived from enjoyment during monitoring and from bodily/emotional changes
Choo et al. (27)	Korea	Phenomenological approach; focus groups	29 community residents in Seoul	To assess the feasibility and acceptability of the “My Heart HELP” mobile application	Three themes: (i) comprehensibility; (ii) elements to remove; (iii) challenges
Chu et al. (32)	USA	Qualitative descriptive study; semi-structured interviews	14 patients enrolled in remote monitoring for hypertension	To evaluate experiences with an RPM-HTN program and identified facilitators and barriers to implementation	Four themes aligned with implementation outcomes: (i)motivation driven by physician recommendation and seeking help for BP management; (ii)telephone support from nurses and pharmacists was satisfactory, though advice was sometimes duplicative; (iii) patients improved understanding and management of hypertension, yet some questioned home BP-monitor accuracy; (iv) convenience of remote monitoring facilitated use, whereas work conflicts and forgetfulness were barriers
Foreman et al. (45)	USA	Grounded theory	17 pregnant individuals with hypertensive disorders of pregnancy who identified as Black/African American/African	To explore facilitators and barriers to using digital tools for self-management of hypertensive disorders in pregnancy and how to culturally tailor such interventions	Seven themes: (i) robust support systems; (ii) bodily autonomy; (iii) trust in healthcare providers; (iv) technology use during pregnancy; (v) negative or discriminatory experiences with providers; (vi) difficulties with diet and comorbidity management; (vii) challenges in self-care and sustaining motivation
Islam et al. (30)	Australia	Phenomenological approach; semi-structured interviews	8 cardiovascular clinicians and 4 patients healthy lifestyles	To investigate views on lifestyle mHealth apps and identified facilitators and barriers to using them to support	Two themes: (i) facilitators; (ii) barriers
Lao et al. (38)	Chinese Macao	Qualitative descriptive study; semi-structured interviews	10 Chinese patients after PCI	To examine usability and satisfaction with a smartphone-based cardiac-rehabilitation app (mCR app) and assessed feasibility in phase-II cardiac rehab in Macao	Four themes: (i) feasibility of the mCR app; (ii) benefits of using the mCR app; (iii) facilitating better hospital care; (iv) suggestions for improving the mCR app
Madujibeya et al. (34)	USA	Qualitative descriptive study; semi-structured interviews	23 patients with HF	To explore real-world experiences using a commercial mHealth app for HF self-care	Five themes: (i) functions that enhance HF self-care; (ii) perceived benefits; (iii) challenges in using the app for self-care; (iv) facilitators; (v) suggested improvements
Neumann et al. (33)	Germany	Phenomenological approach; semi-structured interviews	83 HF patients	To investigate expectations, user experiences, and usage behaviors with a self-care app, including initial and longer-term use	Five themes: (i) expectations; (ii) perceived usability and benefits; (iii) usage behaviors and experiences; (iv) HF self-care; (v) social influences
Schmaderer et al. (40)	USA	Phenomenological approach; semi-structured interviews	10 HF patients	To explore experiences using a self-management mHealth application	Four themes: (i) I did not realize before—now I know; (ii) Focusing on my health makes me feel good; (iii) I am the leader of my care team; (iv) My health is improving

**Table 3:** Descriptive themes and sub-themes derived via meta-aggregation, mapped to the five synthesized findings.

Descriptive themes	Sub-themes
Positive effects of DHTs	Illness perceptions and emotional regulation; Activation and optimization of rehabilitation behavior; Strengthening self-management self-efficacy
Barriers to adoption and sustained use of DHTs	Usability and digital literacy barriers; Environmental and interactional barriers; Economic and social factors
Optimization needs and design priorities for DHTs	Personalisation and plain-language functional design; Coordinated adaptation of support systems; Safety and continuity of use;
Central role of professional support in DHTs implementation	Safety assurance; Fostering trust and digital health literacy
Culture- and disease-specific needs	Influence of cultural background on DHTs engagement; Disease-specific requirements for differentiated tool design

### 3.1 Theme 1: positive effects of DHTs

#### 3.1.1 Illness perceptions and emotional regulation

Some patients reported highly positive experiences with immersive virtual reality (VR). By presenting diverse scenes (e.g., forests, beaches) for people with heart failure, VR reframed rehabilitation from “monotonous repetition” to “travel-like exploration,” increasing acceptance and reducing anxiety “The experience exceeded my expectations; … It was interesting to see places I did not know… making the session feel more like an exploration…” (26). Smartphone applications provided education and peer support, alleviating loneliness and fear “…helps to talk to people who are going through the same stuff because family they do not always understand” (35). Some patients described a dampened sense of time that reduced aversion to prolonged exposure. Interactive guidance during telerehabilitation helped them focus on the task “… Time flies without you realizing it…; …Virtual reality makes you not think about the passing of time…; When you follow the video, you disconnect…” (26). Mobile apps and conversational agents delivered plain-language explanations that addressed knowledge gaps and, in turn, reduced anxiety. For example, patients with heart failure used app animations to understand reduced ejection fraction “maybe… reminder about a particular symptom that might occur that would require attention… I’ve got a pacemaker… I’ve got a couple of stents…; …would rather use an app, which would provide them ‘assurance’ that they are taking their medication” (35). After viewing educational videos distinguishing paroxysmal from permanent atrial fibrillation, patients reported less anxiety “having the ability to monitor their heart rhythm … can be reassuring … and to better manage their symptoms and anxiety” (29). This sub-theme highlights that DHTs can significantly improve the subjective illness experience by reducing anxiety and fostering positive engagement. Therefore, effective interventions should integrate emotional and cognitive support mechanisms alongside clinical monitoring to enhance holistic patient care.

#### 3.1.2 Activation and optimization of rehabilitation behavior

Some patients reported that DHTs reduced constraints of time and place, enabling home-based training and supporting maintenance of rehabilitation routines. “It easily takes two, two and a half hours if you do not do the exercise at home and therefore, I think this is great, because it cuts the timeframe” (36). In-app prompts and progress feedback helped sustain long-term motivation. “I think it helped me stay motivated and made the sessions feel less like a chore; it feels almost as though you are pedaling with less effort because you are psychologically encouraged to follow the music and everything around you” (26), “Yes, I would like to continue, but my exercise period is over, I understand. But then it is good that I have already established a routine to continue on my own” (36). These findings emphasize the DHTs should provide positive feedback, facilitating sustained adherence to rehabilitation protocols, a key component of personalized and effective patient-centered care.

#### 3.1.3 Strengthening self-management self-efficacy

Some patients reported that DHTs consolidate disease-related information (e.g., symptoms, diet) in one place, reducing the fragmentation and noise of general online sources and enhancing credibility. “I have everything in one particular place, I know because the app is geared specifically to heart issues, I know that I can trust the information on the app” (35). These applications also support precise self-monitoring by enabling recording of blood pressure, weight, and other indicators, thereby helping patients and clinicians track trends. “I measure my blood pressure twice a week; the platform prompts me to upload data at least once weekly, and I now have a clearer sense of my blood-pressure pattern” (39). Some patients added that goal setting and feedback incentives facilitated lifestyle change. “With that medication reminder and then weighing myself every day to see if I was retaining water, it helped me a lot…” (40). By centralizing information and facilitating self-monitoring, DHTs empower patients, increasing their confidence in managing their condition and provide actionable insights.

### 3.2 Theme 2: barriers to adoption and sustained use of DHTs

#### 3.2.1 Usability and digital literacy barriers

Patients reported physical discomfort and performance issues with hardware-dependent tools (e.g., virtual reality), which impaired usability. “The headset sometimes bothered me a bit;… it’s often out of focus, never completely sharp…” (26), “My phone was too old, and it does not support apps like this” (28). These systems had software glitches, connectivity problems, and cumbersome workflows that hindered use. “Screening… was a bit cumbersome, it was another element to the whole start-up, so it was just another layer of… hassle” (44). Multi-device configurations (e.g., the PATHway system requiring both a computer and an Xbox) demand composite skills, including device configuration, troubleshooting, and procedural memory. Yet most CVD patients are older adults who lack experience with complex technologies “I found that there was too many bits… you had a computer, you had the X-Box… and everything had to be plugged in a certain way and you did not have enough power sockets. Then you kept running out of power and there were cables trailing. So, it was a bit cumbersome” (44). These technical challenges were often exacerbated by limited digital health literacy. Some older adults struggled to operate devices and applications. “I’m not used to using apps, not even using a cell phone, so figuring out the AI was really difficult at first. It took me two visits to the hospital to really learn how to use it… My son helped me too” (28). In addition, lower educational attainment may foster misconceptions about technology that hinder use. “I only attended school through third grade. With this high-tech app, I do not know how to keep using it” (31). Fundamentally, these challenges reflect an inadequate translation of professional information into accessible, plain language within digital tools. To overcome these barriers, DHTs should be designed with simplicity in mind, ensuring that they are intuitive, easy to use, and compatible with a range of devices.

#### 3.2.2 Environmental and interactional barriers

Some middle-aged and younger adults reported less frequent data entry due to competing work demands. “When I first enrolled, I uploaded my blood pressure daily; now that work is busy, I log it every 3 days” (39). Comorbid conditions and age-related visual decline also impeded use. “The starting level was too high for me; I am 75 and I have never used a computer; Sometimes my body was very painful… I did not feel up to it at the time” (44). “My eyesight is poor. Even with large fonts, the platform has too many sections and not all display on my screen; I sometimes need my children’s help” (39). The design of DHTs must consider patients’ daily environments and health conditions, providing flexibility to accommodate varying schedules and physical capabilities. Features such as voice-assisted data entry or automated reminders can help overcome these interactional barriers, ensuring that DHTs remain useful and accessible for all patients, regardless of their circumstances.

#### 3.2.3 Economic and social factors

Some patients expressed cost-related concerns about using DHTs. “But before that I will ask, all these, right, will you charge to us? Because my husband is not working, I’m not working myself …” (42). “It is free now, but will it remain free? If not, can health insurance reimburse the fees?” (41). Patients in remote areas, or those with limited hardware support, often face practical constraints. “Discontinuation of Internet access at home, loss of program information after changing the phone, and device failure” (37). Therefore, policymakers and healthcare providers should prioritize affordable, low-cost DHTs to ensure equitable access. Cost should not be a barrier to care, and health insurance reimbursement policies should cover DHTs to avoid exacerbating health inequities.

### 3.3 Theme 3: optimization needs and design priorities for DHTs

#### 3.3.1 Personalisation and plain-language functional design

Patients emphasized the need for tailoring by disease type, stage, baseline knowledge, and personal preferences. Generic push notifications provided little disease-specific value. “…they wanted targeted information that was relevant to their disease status … and type of AF” (29). “Vague ‘how are you’ messages are not useful; I need professional, condition-specific information” (31). Older adults called for age-friendly design. Complex data entry, multiple steps, and non-essential features reduced willingness to use. “There are too many fields and screens; the selector is small, and if I forget to save I must re-enter everything” (41). Others reported excessive jargon and abbreviations in push content, limiting comprehension. “It is full of abbreviations and obscure terms” (31). A subset lacked intrinsic motivation and perceived low utility. “I am not convinced the management app helps beyond my medications” (41). The strong demand for personalized and plain-language design highlights a gap between current DHTs offerings and patient needs. This points to the necessity of user-centered design processes that actively involve patients, especially older adults, in co-creating tools that are both relevant and accessible.

#### 3.3.2 Coordinated adaptation of support systems

Patients emphasized that problems are inevitable during use and called for stronger professional and social support to reduce barriers. “I messaged the nurse about stomach discomfort after taking my medication. She suggested ways to relieve it, reminded me of possible side effects, and advised seeing a gastroenterologist if symptoms persisted” (38). Participants wanted clinicians to access longitudinal app data to inform decisions, optimize treatment plans, and streamline consultations. “If the cardiologist could review 6 months of my blood-pressure and activity data in the app, that would be ideal” (35). Integrating social support helped family members understand the illness and enabled peer-to-peer communication. “Having a community means I can ask others, ‘Do you have this symptom? What happened?’ when symptoms start” (35). Several patients reported insufficient onboarding and troubleshooting support, leading to over-reliance on family members. “At the start, a 15 min onboarding session to demonstrate set-up and audio would help” (36). “My daughter is good at IT; I ask her, and she helps me” (44). So, DHTs should be integrated into a broader ecosystem of care that includes both clinical professionals and informal support systems like family and peer networks. This calls for policies that facilitate seamless communication and data sharing between patients, healthcare providers, and caregivers, ensuring that DHTs are not isolated but part of a holistic, patient-centered care model.

#### 3.3.3 Safety and continuity of use

Some patients expressed concerns about potential disclosure of personal health information when using DHTs. “I recognize that some data sharing is unavoidable, but the possibility of online leakage and misuse makes me uncomfortable” (43). Others recommended extending the service period to support the internalization of health behaviors as daily habits. “If all functions of mCR app can be offered … for 1 year after hospital discharge, it’ll be much better for patient’s self-care as some medications (dual antiplatelet therapy) should be adhered for 1 year” (38). Therefore, two fundamental nursing strategies are crucial for DHTs success: fostering trust through proactive education on data security to empower patients, and securing long-term engagement by advocating for DHTs designs that correspond to clinical pathways and support behavior internalization.

### 3.4 Theme 4: central role of professional support in DHTs implementation

#### 3.4.1 Safety assurance

Patients reported that clinicians’ real-time supervision and professional judgment mitigated risks associated with using DHTs. For example, during telerehabilitation, physiotherapists’ real-time monitoring (e.g., heart rate, movement quality) provided reassurance. “It’s a sense of security, that someone who actually has in-depth expertise and knows what I need to exercise in terms of fitness and strength, both how much and what is best for me, because I’m not fully aware of that” (36). Nurses also monitored app data and intervened proactively to reduce deterioration risk. “I used to just keep papers on the counter with my weight and I like this (OnTrack) better and if I see my weight go up, I send a note to a nurse and there is help” (34). This highlights the importance of integrating professional oversight into the use of DHTs. Therefore, DHTs should include features that facilitate healthcare providers access to real-time data, fostering collaboration between patients and their healthcare teams, a critical aspect of patient-centered care.

#### 3.4.2 Fostering trust and digital health literacy

Clinicians’ endorsement of DHTs enhances patients’ trust and willingness to engage with the program. “If my treating physician emphasizes the importance of rehabilitation and recommends downloading an app for self-monitoring because it helps, I will use it” (31). Nurses deliver structured onboarding and hands-on training to support patients with limited digital health literacy; when needed, they also train relatives or younger family members. “This mCR app suits me, but older adults with limited literacy or unfamiliar with smartphones may have difficulties; nurses can teach relatives or younger family members how to use it” (38). Professional support is not merely an adjunct but a foundational component for successful DHT implementation. Healthcare professionals act as gatekeepers of trust, safety, and knowledge. This highlights the critical need to equip clinical staff with the skills to effectively support patients’ digital health journeys, integrating these new tools into standard care pathways.

### 3.5 Theme 5: culture- and disease-specific needs

#### 3.5.1 Influence of cultural background on DHTs engagement

East Asian (China, Hong Kong, China, and Macao, China): Family involvement was a significant factor. Patients from these regions frequently requested that their caregivers, particularly family members, have access to app data. This reflects the culturally ingrained value placed on family support in healthcare. For example, one participant from China noted, “Could my daughter view my blood pressure on her phone? I am often home alone, and my daughter works in another city; real-time access would ease her concerns” (38). Such requests were more common among Chinese participants compared to other regions.

Western Countries (USA, Ireland): While family involvement was also noted, the engagement was more individualistic. Patients in these regions expressed a preference for clinicians to have access to digital health data to provide ongoing support. The desire for clinician involvement reflects a more clinical-centered approach to care, as exemplified by a participant in Ireland who stated, “If my treating physician emphasizes the importance of rehabilitation and recommends downloading an app for self-monitoring because it helps, I will use it” (31).

LMICS (Africa and African-American Communities): In specific populations such as Black pregnant women in the USA, cultural beliefs surrounding race and ethnicity influenced trust in healthcare providers. Participants in these studies expressed a preference for race-concordant clinicians, believing they would understand their lived experiences better. One participant shared, “A Black female physician would understand me; I would be more willing to confide in her” (44). This highlights how cultural identity can influence trust and the willingness to engage with DHTs. So culturally competent nursing strategies for DHT implementation include: (1) promote family-integrated care in East Asian contexts to leverage collective support, (2) strengthen the clinician-patient alliance in Western settings by highlighting professional monitoring, and (3) ensure cultural safety and address representation for marginalized groups to build foundational trust.

#### 3.5.2 Disease-specific requirements for differentiated tool design

Patients with heart failure emphasized the importance of fluid management and daily weight monitoring. However, patients in Western countries also requested real-time feedback from healthcare professionals, such as “If the cardiologist could review 6 months of my blood-pressure and activity data in the app, that would be ideal” (35). Conversely, Chinese participants focused more on practical, day-to-day tracking without as much emphasis on professional monitoring.

Patients with AF in the USA and Europe prioritized heart-rate monitoring and stroke-risk alerts in their DHTs. The distinction between paroxysmal and permanent AF was often requested, with one patient noting, “When my AF symptoms occur, the program is more useful; I need information tailored to my status and AF type” (28). While these concerns were universal, USA patients tended to focus more on educational content regarding medication adherence and stroke prevention, whereas European patients expressed a need for emotional and psychological support during episodes.

In LMICs, where hypertension is a major health concern, patients preferred simpler, more accessible DHTs that could be used by individuals with lower health literacy. For instance, one participant from China emphasized, “I knew I should eat low salt and low fat, but I did not know what those terms actually meant” (38). These problems reflect effective DHTs must be disease-specific, offering tools tailored to the unique management needs of each condition. Additionally, they should be adaptable to local healthcare systems, patient literacy levels, and cultural practices.

## 4 Discussion

This qualitative meta-synthesis integrates related evidence to distill five core insights on DHTs for patients with cardiovascular disease. DHTs show substantial potential to strengthen emotional self-regulation, support rehabilitative behaviors, and enhance self-efficacy; however, uptake is constrained by multilevel barriers spanning usability, digital literacy, contextual demands of everyday life, and socioeconomic constraints. Together, these findings argue for multidimensional personalization, embedded professional support, and culturally adapted design to realize patient-centered implementation and align with the broader digital transformation of cardiovascular care. Our review offers a more comprehensive perspective than prior research that has primarily focused on clinical effectiveness.

A significant regional difference was observed in how cultural values influence patient engagement. In China, Hong Kong, China and Macao, China, patients demonstrated a strong preference for family involvement in managing their health via digital tools, reflecting the cultural value placed on family-centered healthcare. Research (46) shows that in these regions, DHTs should prioritize user-centered designs that integrate traditional values and address differences between patients and caregivers. This contrasts with Western countries (USA, Europe), where patients expressed a stronger preference for clinician engagement with digital tools. Patients in these regions valued healthcare professionals’ involvement in interpreting health data and providing continuous support. For instance, a national survey (47) of 352 mental health professionals in the USA found that 74.2% of respondents preferred to integrate DHTs into clinical practice. However, disconfirming evidence emerged in some populations, particularly among African-American patients in the USA, where a lack of trust in technology was reported. Some patients were skeptical about the accuracy and reliability of DHTs, especially when these tools were not directly linked to trusted healthcare providers. This finding highlights that cultural identity and healthcare system trust significantly impact technology acceptance. Related studies (48–50) indicate that African-American communities historically face mistrust in healthcare systems due to past discriminatory practices, which further compounds their reluctance toward adopting new technologies. This suggests that DHTs should not only account for digital literacy but also the trust patients have in the tools they use. By understanding these cultural and systemic factors, DHTs can be better designed to ensure broader acceptance and engagement.

Disease-specific needs also reveal important contextual nuances in HF. Across Western and Asian settings, patients consistently emphasize tools for fluid management (e.g., tailored fluid limits), daily weight monitoring, and tracking worsening symptoms—features reflected in HF self-care guidance and in reviews of HF self-management apps (51–53). However, preferences for clinician involvement vary by region. In Western health systems, qualitative studies of HF telemonitoring report that patients value real-time feedback and clinical staff oversight when sharing weight and symptom data (35, 54). By contrast, studies (55, 56) from China frequently highlight app-enabled, WeChat-based self-management models that emphasize independent daily tracking with intermittent professional input—an approach many patients find acceptable and effective for routine management.

Beyond cultural and disease-specific factors, regional disparities in economic conditions and technological infrastructure also presented significant barriers to the adoption of DHTs. In LMICs, patients faced significant barriers to engaging with these digital tools due to poor infrastructure, such as unreliable internet access and limited access to the required hardware (57). These barriers highlight the systemic limitations that hinder the success of DHTs, despite their potential to improve disease management. Disconfirming evidence from these regions suggested that remote monitoring and self-reported data were often insufficient for effectively managing acute conditions. For acute fluid retention in HF, self-report–dominant remote programs showed neutral effects (e.g., TELE-HF) (58), whereas approaches coupling continuous physiologic data with structured clinician oversight (e.g., TIM-HF2; PA-pressure monitoring) reduced events—highlighting the need for real-time clinical review in high-risk contexts (59, 60).

The internal design of DHTs—particularly their degree of personalization—profoundly influences their adoption and impact. In China, where family involvement is crucial, tools should incorporate features that allow caregivers to access health data. Meanwhile, in Western countries, the focus should be on clinician-centered features that enable healthcare providers to monitor progress in real-time. Moreover, as older adults are prevalent in many CVD patient populations, age-friendly design remains crucial for improving usability, particularly in Western populations where digital health literacy can be a barrier. However, disconfirming evidence arose from both European and North American studies, where patients voiced frustrations about receiving irrelevant or overly complex information (61). These findings suggest that while personalization is important, it must be executed carefully to avoid overwhelming users. Overly generic content and complex user interfaces can lead to disengagement, indicating that DHTs must strike a balance between personalization and simplicity.

Professional support is universally recognized as critical to the success of DHTs. In Western countries, patients typically prefer continuous engagement with healthcare providers to ensure effective use of these tools (62–64). In contrast, patients in Asian countries, such as China and Korea, demonstrate higher self-reliance, favoring tools that require minimal clinician involvement. Despite these cultural differences, clinician training emerges as a common systemic barrier (65). Even in high-income countries, many clinicians are unfamiliar with the digital tools used by their patients, limiting their ability to effectively interpret the digital health data (66, 67). This knowledge gap impedes the full potential of DHTs. Additionally, the lack of integration between DHTs and clinical workflows further hinders their successful implementation (68). These challenges underscore the need for comprehensive education programs for healthcare professionals to ensure effective data sharing, improve clinician familiarity with digital tools, and promote personalized care.

## 5 Limitations

This review focuses on qualitative studies, which provide in-depth insights but may limit generalisability due to the predominance of studies conducted in China, the USA, and Europe. Consequently, populations in regions such as Sub-Saharan Africa and Latin America are underrepresented. Cultural and healthcare system differences in these regions may influence patients’ engagement with and experiences of DHTs.

Although the analysis was conducted rigorously using Joanna Briggs Critical Assessment Tool and master tutor consultation to minimize bias, the inherent subjectivity of qualitative research remains a consideration.

Furthermore, the included studies varied in sample sizes and the duration of DHTs usage, which may influence the findings. The range of digital tools and platforms used across studies adds another layer of variability, though this diversity reflects real-world applications of DHTs in different contexts.
